# Adolescent Pregnancy in South Asia: A Pooled Analysis of Demographic and Health Surveys

**DOI:** 10.3390/ijerph20126099

**Published:** 2023-06-10

**Authors:** Samikshya Poudel, Timothy Dobbins, Husna Razee, Blessing Akombi-Inyang

**Affiliations:** School of Population Health, University of New South Wales, Sydney, NSW 2052, Australia

**Keywords:** adolescent pregnancy, teenage pregnancy, risk factor, South Asia, Afghanistan, Bangladesh, India, Maldives, Nepal, Pakistan

## Abstract

Adolescent pregnancy has important health and social implications. Despite the availability of nationally representative household survey data, there are limited studies that analyze factors associated with adolescent pregnancy across countries of South Asia. This study aimed to identify factors associated with adolescent pregnancy across South Asia. This study used the most recent Demographic and Health Survey (DHS) data from six countries in South Asia: Afghanistan, Bangladesh, India, the Maldives, Nepal, and Pakistan. Pooled individual record data from 20,828 ever-married women aged 15–19 years were used for the analysis. Multivariable logistic regression analysis, informed by the World Health Organization framework on social determinants of health, was performed to examine factors associated with adolescent pregnancy. Adolescent pregnancy was highest in Afghanistan compared to Bangladesh, Nepal, Pakistan, India, and the Maldives. Multivariable analyses confirmed that being from a poor household or male-headed household, increasing maternal age, having no access to newspapers, and having no knowledge of family planning were significantly associated with adolescent pregnancy. The use or intention to use contraceptives was protective against adolescent pregnancy. To reduce adolescent pregnancy in South Asia, interventions targeting adolescents from poor households with limited access to mass media should be considered, especially those from households with an existing patriarchal structure.

## 1. Introduction

Approximately 17% of the world population is adolescents [1]. While adolescence is considered as an important phase for physical and psychological growth in the human life span, it also tends to be combined with various risk-taking behaviors, including early pregnancy [2]. The World Health Organization (WHO) defines adolescent pregnancy as the occurrence of pregnancy in girls aged 10–19 years [3]. Research has shown that adolescent pregnancy is associated with various health risks, including low birth weight [4], prematurity [5], stillbirth [6], congenital anomaly [7], infant death syndrome [4], and adverse fatal neonatal health issues [8]. In addition to adverse physical consequences, adolescent mothers are predisposed to different forms of psychological issues such as depression, anxiety, conduct disorders, and post-traumatic stress disorders [9,10,11]. Research has shown that children born to adolescent mothers experience health and social complexities such as a lower level of cognitive stimulation, behavioral problems, lower academic performance, and an increased risk of dropping out of school [12]. Early adolescent childbearing (before the age of 16) has been linked with a greater likelihood of having a complicated birth [13].

The burden of adolescent pregnancy in developing countries is substantially higher than that reported in developed countries [6]. Globally, 12 million adolescent girls aged 15–19 years give birth every year, and about 777,000 girls aged less than 15 years give birth in developing regions [6]. In addition, an estimated 3.9 million adolescent girls undergo unsafe abortions every year, resulting in an increased risk of health complications or death [6]. South Asia has reported the second highest rate of adolescent pregnancy after Sub-Saharan Africa [14,15]. South Asian countries have different minimum legal marriage ages for girls (Nepal: minimum 20 years; Bhutan, Bangladesh, India, the Maldives, and Sri Lanka: minimum 18 years; Afghanistan: minimum 16 years; Pakistan: minimum 16 years, except for in Sindh province, where the minimum age for marriage is 18 years) [16,17].

Despite the high burden of adolescent pregnancy in South Asia, studies that identify factors associated with adolescent pregnancy are limited. Existing literature on factors associated with adolescent pregnancy in South Asia are either country specific or community based with a small sample size [15,18,19,20,21,22,23], which makes the generalizability of findings to the wider South Asian population quite low. Future regional studies that utilize pooled samples from South Asian countries may be beneficial for bridging the knowledge gaps, which is essential for formulating regional-level programs and policies to prevent adolescent pregnancy. This study, therefore, aimed to examine factors associated with adolescent pregnancy in South Asia by utilizing the most recent Demographic and Health Survey data from six South Asian countries. Findings from this study can assist public health practitioners, health researchers, and policymakers in formulating and implementing socio-cultural interventions to prevent adolescent pregnancy in South Asia. Findings from this study can also be beneficial in identifying consistent factors associated with adolescent pregnancy across the countries of South Asia, allowing for more comprehensive regional-level programs.

## 2. Materials and Methods

### 2.1. Data Source

The Demographic and Health Survey (DHS) is a nationally representative household survey which collects data for a wide range of monitoring and impact evaluation indicators in the areas of population, health, and nutrition [24]. The survey is typically conducted in participating countries about once every 5 years to allow comparisons over time. The DHS Program is funded by the U.S. Agency for International Development and implemented by Inner City Fund [25]. The DHS collects data from participating countries using standard-model questionnaires that are comparable across countries. The survey employs a stratified multi-stage cluster design in the selection of samples. Further details on the sampling strategy can be found in the corresponding DHS final report [26,27,28,29,30,31].

The data for this study were extracted from the most recent DHSs of six countries in South Asia: Afghanistan (DHS 2017), Pakistan (DHS 2017–2018), Nepal (DHS 2016), India (DHS 2019–2021), the Maldives (DHS 2016–2017), and Bangladesh (DHS 2017–2018). Sri Lanka and Bhutan were not included in this analysis because the last DHS conducted in Sri Lanka was in 1987, while Bhutan has never conducted a DHS. Individual records for women aged 15–19 years from the six DHS countries were extracted. The sample women from India, the Maldives, and Nepal included ever-married women and those that have never married, whereas the sample women from Afghanistan, Bangladesh, and Pakistan included only those who have ever been married. Ever-married women include women who are currently married, living with partner, widowed, divorced, and no longer living together with their partners, whereas never-married women are those who have never been in a marital relationship. For the purpose of this study, the analyses were restricted to ever-married adolescent women aged 15–19 years. 

### 2.2. Outcome Measure

The outcome measure for this study was adolescent pregnancy, defined as pregnancy among adolescent girls aged 15–19 years [26,27,28,29,30]. The study outcome ‘adolescent pregnancy’ was reported as a binary variable and coded as 1, whereas ‘no pregnancy’ was coded as 0.

### 2.3. Explanatory Variables

The explanatory variables used in this analysis were classified based on the modified WHO conceptual framework for social determinants of health [32]. According to the framework, the variables were grouped into four main categories: country- and community-level socio-economic factors, household-level socio-economic factors, individual-level socio-economic factors, and individual-level behavioral and social conditions.

Country- and community-level factors were geographic and administrative location of residence, which included DHS country of survey and type of residence. Household-level factors were household wealth index and sex of household head (female/male). Household wealth index serves as an indicator of wealth that is consistent with expenditure and income measures. It is represented as a score of household assets via the principle components analysis method (PCA) [33]. The index is categorized into five national-level wealth quintiles: richest, richer, middle, poorer, and poorest. 

Individual-level socio-economic factors were the women’s age (15 to 19 years), education status (secondary or higher/primary/no education), and literacy status (literate/not literate). Behavioral and social variables consisted of access to radio (yes/no), access to television (yes/no), access to newspapers (yes/no), heard of family planning on radio (yes/no), heard of family planning on television (yes/no), heard of family planning in newspaper (yes/no), and intention to use contraceptives (does not intend to use/currently using/intends to use later).

### 2.4. Statistical Analysis

Data were analyzed using STATA (version 14.1) (Stata Corp, College Station, TX, USA). In the descriptive analyses, rates of adolescent pregnancy with 95% confidence intervals (CIs) were presented per 1000 women by socio-demographic characteristics. We then estimated unadjusted and adjusted odds ratios (ORs) with 95% CI. All analyses were adjusted for the complex survey design using the “svy” commands in STATA.

A four-stage modified WHO conceptual framework for action on the social determinants of health was used to determine the factors associated with adolescent pregnancy in South Asia. Initially, all the factors were assessed for multicollinearity using variance inflation factors. All variance inflation factors were less than three, suggesting a low degree of multicollinearity [34]. We then performed univariate logistic regression to examine the unadjusted association between each exploratory variable and study outcome followed by multivariable logistic regression. At the first stage of multivariable logistic regression, country- and community-level factors were analyzed with a manual backward elimination process to remove non-significant variables (model 1). In the second stage, household-level factors were added into model 1, and a manual backward elimination process was applied to remove non-significant variables (model 2). A similar process was repeated in the third and the fourth stages that analyzed individual-level factors with model 2 (model 3) and individual/social behavioral factors with model 3 (model 4), respectively, with a backward elimination process to remove statistically non-significant factors. The significance level was set at 0.05, and variables significantly associated with adolescent pregnancy in model 4 are reported in this manuscript. 

### 2.5. Ethical Consideration

This study was based on secondary data analysis; hence, no ethical approval was required. However, permission to use the datasets from all six South Asian countries was sought and obtained from MEASURE DHS for this study. Data were collected by interviewers using standard questionnaires. Consent statements were read to all eligible respondents, and informed verbal consent from each participant was confirmed by signature of the interviewer.

## 3. Results

### 3.1. Characteristics of Study Population

Table 1 shows the characteristics of the study population. Of the married adolescent women in the sample, 74.8% were from India, whereas less than 1% were from the Maldives. Of the adolescent girls, 81% were from rural areas, and 28.5% belonged to the poorest households. Seventeen percent of the adolescent girls were uneducated. Of the adolescent girls, 85.5% were from a male-headed household. Nearly half (44.5%) of the total adolescent girls had no intention to ever use any means of contraception. Access to the radio and newspapers was low at 14.6% and 20.6%, respectively.

### 3.2. Adolescent Pregnancy

Table 2 shows the adolescent pregnancy rate per 1000 ever-married women for six countries in South Asia. Adolescent pregnancy ranged from 395 (95% CI: 387, 404) per 1000 ever-married women in the Maldives to 711 (95% CI: 700, 723) per 1000 ever-married women in Afghanistan.

Table 3 presents adolescent pregnancy in ever-married women by country and socio-demographic characteristics. Women with no education were more likely to report a higher rate of adolescent pregnancy in Afghanistan, Bangladesh, India, and Pakistan compared to those with a higher level of education. Adolescent pregnancy was also significantly higher among illiterate women compared to literate woman in Bangladesh.

Table 4 shows the unadjusted and adjusted odds ratios (OR) for factors associated with adolescent pregnancy in South Asia. The adolescent pregnancy rate was higher in Bangladesh (aOR 2.60, 95% CI 2.26, 2.99), Afghanistan (aOR 2.32, 95% CI 1.91, 2.82), Nepal (aOR 2.53, 95% CI 2.06, 3.11), and Pakistan (aOR 1.43, 95% CI 1.13, 1.80) compared to in India. Women from households rated as middle, poorer, and poorest on the wealth index had higher odds of experiencing adolescent pregnancy compared to those from a wealthy household. Adolescent women living in households with a male head were more prone to experiencing pregnancy compared to adolescent women living in a household with a female head (aOR 1.15, 95% CI 1.03, 1.29). Adolescent girls who were older (16 years, 17 years, 18 years, and 19 years old) were more susceptible to pregnancies than 15-year-old girls. In addition, adolescent women reporting no access to newspapers and magazines (aOR 1.35, 95% CI 1.21, 1.50) and those who gained no knowledge of family planning through newspapers and magazines (aOR 1.18, 95% CI 1.06, 1.31) were more likely to experience early pregnancies than those who had actual access to those media. Finally, women who were currently using contraceptives and those who intended to use them later in life were less likely to experience adolescent pregnancy compared to those who did not intend to use contraceptives ever.

## 4. Discussion

Despite adolescent health programs and policies [35,36], the adolescent pregnancy rate is still high in South Asia. Our study identified factors associated with adolescent pregnancy across countries in South Asia. Having a low household wealth index, residing in a male-headed household, increasing age, and failure to read the newspaper or hear about family planning via the newspaper were the identified factors associated with adolescent pregnancy in South Asian countries. 

In this study, amongst the South Asian countries analyzed, the adolescent pregnancy rate was highest in Afghanistan. This is in line with previous studies that reported high unwanted pregnancy rates among adolescent girls aged < 18 years in Afghanistan [37]. The higher odds of adolescent pregnancy in Afghanistan were reported to be due to multiple factors, including socio-cultural beliefs, lack of education and employment opportunities, residence in geographically resource-poor settings, and early sexual debut [38]. Interventions targeting these multiple factors could assist in reducing adolescent pregnancy rates in Afghanistan. One such intervention is the provision of community-based culturally sensitive comprehensive sexuality education (CSE) to adolescents residing in geographically resource-poor settings. CSE, which is a curriculum-based process of teaching and learning about the cognitive, emotional, physical, and social aspects of sexuality, has proven to be effective in reducing sexual debut age and, consequently, early pregnancy among adolescents [39]. CSE could be streamlined to consider the unique socio-cultural beliefs within Afghanistan which predispose adolescents to early pregnancy. In contrast, the Maldives reported significantly lower odds of adolescent pregnancy compared to other countries within South Asia. Lower rates of early marriage and higher literacy rates have previously been discussed as possible factors linked to delayed pregnancy and childbirth among adolescents in the Maldives [40]. Additionally, the latest World Health Organization factsheet showed that women surpass men in employment, school enrollment, and use of social media in the Maldives [41], which may contribute to lowering the rate of adolescent pregnancy. However, the protective effect against adolescent pregnancy among literate women in this study did not differ significantly.

This study reported that adolescents from poor households are more susceptible to pregnancy than those from more affluent households. This finding is consistent with previous research conducted in both resource-poor and resource-affluent settings [42,43]. Adolescents with lower socio-economic status may have lesser exposure and access to contraceptives [44]. Research has shown a higher use of contraceptives among adolescents from resource-affluent households [45], thus, preventing pregnancy. In addition, culture and the normative pressure of the dowry system are still prevalent in various parts of South Asia. In the dowry system, the family of the groom demands durable goods, cash, and real or movable property as a condition of marriage. Furthermore, young married girls would like to become pregnant earlier as a strategy to prevent their husband from getting other wives [46]. Parents of young girls with poor economic backgrounds tend to achieve marriage for their daughters when the daughters are younger to avoid having to pay the higher dowry associated with an older age, thus leading to early pregnancy [47,48]. A few pieces of research from the Muslim-dominated countries of South Asia such as Afghanistan, Bangladesh, and Pakistan have shown higher odds of child marriage, which increases the odds of adolescent pregnancy. One possible reason for this is that Islam promotes child marriage, with no restriction imposed on age of marriage [40,49,50].

In this study, adolescent girls residing in male-headed households were more prone to adolescent pregnancy compared to adolescents residing in a female-headed household. Due to existing patriarchal norms within South Asia, an adolescent has limited autonomy in household decision making, including decisions relating to childbearing and the use of contraception [51], which might increase the likelihood of pregnancy.

In our study, we reported that, as adolescent girls increase in age, so they are more likely to get pregnant. Numerous studies from low- and middle-income settings have reported an increase in adolescent pregnancy with increased age [52,53,54,55]. Older adolescent girls tend to be more capable of performing household chores unsupervised, which makes them suitable as young brides [53], especially in cultures where early marriage is prevalent, thus predisposing them to early pregnancy. However, contrary to our findings, population-based studies conducted in Uganda [56] and Indonesia [57] showed no significant association between increase in age and pregnancy among adolescents. 

The protective effect of contraceptive use or intention to use contraceptives against adolescent pregnancy reported in this study is in line with a previous population-based study conducted in East Africa [58]. It could be insinuated that adolescent girls who currently use contraceptives or intend to use contraceptives are aware of the adverse effect of early pregnancy and the role of family-planning methods in preventing it. A cross-sectional study by Wang et al. [59] concluded that adolescent girls with poor family-planning knowledge are at a higher risk of pregnancy. Therefore, enhanced knowledge on contraceptives use and their health benefits is needed among adolescents to prevent early and unplanned pregnancy.

The current study also reported the role of the mass media in preventing adolescent pregnancy. The mass media serves as a medium to communicate salient information on adolescent sexual health to the target demographic. Exposure to mass media has been reported to promote health-seeking behavior, enlightenment around the appropriate age for marriage, pregnancy, and childbirth, and the use of contraceptives to reduce adverse health outcomes [60]. In line with our study, previous studies conducted in South Asia reported a positive association between exposure to mass media and reduced occurrence of adolescent pregnancy [15,19,61]. However, in our study mass media was limited to radio, TV, and newspapers. While these forms of mass media are appropriate to spread messages about family planning among adolescents, particularly among those from poorer backgrounds, more modern forms of media such as social media may need to be considered in the twenty-first century. Moreover, knowledge alone is not sufficient for adolescents to change their intention to use contraceptives into actual behavior. Accessibility of contraceptives is essential, and no data were available on this in the dataset used in this study.

Our study had a number of notable strengths. First, the DHS is a large, weighted dataset, and the use of a pooled DHS sample increased the statistical power of the study. Providing stronger evidence by utilizing nationwide pooled data from countries of South Asia is itself an innovative scientific approach. Evidence from this study can bridge existing knowledge gaps by determining consistent factors associated with adolescent pregnancy in South Asia, and the evidence can be beneficial o inform future regional health strategies for the prevention of adolescent pregnancy. Second, the questionnaires used by the DHS are standard across countries, facilitating easy variable comparison across countries. Third, this study has implications for healthcare planners and policymakers designing future reproductive health initiatives aiming to reduce adolescent pregnancy in countries of South Asia. However, our study should be considered in the light of a few limitations. The DHS is a cross-sectional household survey and, therefore, causal relationships between independent variables and the outcome variable (adolescent pregnancy) cannot be established. The inconsistency of the samples across the DHSs from the six different countries resulted in only ever-married adolescent girls aged 15–19 years being included. This might have induced selection bias by not including unmarried women or girls below 15 years of age. A lot of DHS survey questionnaires (including those on women’s education) are based on self-reporting, which may increase response bias. Additionally, the generalizability of findings to the entirety of South Asia might be questioned as our analysis did not include all South Asian countries, excluding those such as Sri Lanka and Bhutan. Factors such as religion and culture, pregnancy desire, and exposure to social media were not included in this pooled study because of a lack of data. Future studies that can incorporate these factors could shed more light on how to reduce adolescent pregnancy. 

### Policy Implications

This study has important policy implications. Cost-effective initiatives aimed at promoting adolescent females’ education and community-based income-generating vocational training incorporated into existing income generation programs can be effective measures for preventing adolescent pregnancy [15]. Future research investigating trends over time and inequality in accessibility and utilization of contraceptives among married adolescent girls in South Asia could shed more light on the formulation of future reproductive health strategies. For a highly patriarchal region such as South Asia, awareness-generating programs around adolescent pregnancy and its longer-term adverse health and social impact aimed at male household heads could be beneficial for reducing adolescent pregnancy rates.

## 5. Conclusions

This study found lower socio-economic status, living in a male-headed household, increasing maternal age, low exposure to newspapers, and low awareness of family planning gained through newspapers to be associated with adolescent pregnancy in South Asia. This study also highlighted the protective effect of contraceptive use in preventing adolescent pregnancy. Therefore, to reduce adolescent pregnancy in South Asia, there is a need to promote education and employment opportunities, especially among economically disadvantaged adolescents. The use of mass media and social media platforms in disseminating reproductive health educational messages could also be effective in improving knowledge on the detrimental effect of adolescent pregnancy among adolescent females, society, and the region.

## Figures and Tables

**Table 1:** Characteristics of study population (*n* = 20,828).

Study Variables	Unweighted	Weighted *	
Country- and community-level factors	*n*	*n*	%
Country of survey			
India	13,760	15,584	74.8
Bangladesh	1951	2063	9.9
Afghanistan	1829	1825	8.8
Maldives	55	43	0.2
Nepal	749	714	3.4
Pakistan	728	600	2.9
Type of residence			
Urban	3479	3944	18.9
Rural	15,593	16,885	81.1
Household-level factors			
Wealth index			
Richest	1439	1660	8.0
Richer	2878	3328	16.0
Middle	3897	4448	21.4
Poorer	5126	5457	26.2
Poorest	5732	5935	28.5
Sex of household head			
Female	2759	3030	14.6
Male	16,313	17,798	85.5
Individual-level factors			
Woman’s age in years			
15	447	549	2.6
16	1079	1269	6.1
17	2283	2588	12.4
18	6691	7166	34.4
19	8572	9256	44.4
Woman’s education			
Higher/secondary	13,319	14,944	71.8
Primary	2281	2437	11.7
No education	3472	3447	16.6
Literacy status (*n* = 20,740)			
Literate	14,133	15,634	75.1
Not literate	4870	5106	24.5
Individual/social behavioral factors			
Access to radio			
Yes	2923	3042	14.6
No	16,148	17,786	85.4
Access to television (*n* = 20,823)			
Yes	11,681	13,049	62.7
No	7387	7774	37.3
Access to newspaper (*n* = 20,823)			
Yes	3753	4294	20.6
No	15,314	16,529	79.4
Heard of family planning on radio (*n* = 20,824)			
Yes	2268	2363	11.4
No	16,799	18,451	88.6
Heard of family planning on television (*n* = 20,826)			
Yes	7989	8960	43.0
No	11,079	11,866	57.0
Heard of family planning in newspaper (*n* = 20,826)			
Yes	4031	4537	21.8
No	15,037	16,289	78.2
Intention to use contraceptives (*n* = 20,825)			
Does not intend to use	8553	9276	44.5
Currently using	5089	5675	27.3
Intends to use later	5425	5874	28.2

* Women’s individual sample weight variable (v005) and primary sampling unit variable (v021) were used to estimate weighted values.

**Table 2:** Adolescent pregnancy rate and 95% confidence intervals per 1000 ever-married women in South Asian countries.

Country of Survey	Rate	95% CI
Afghanistan	711	(700, 723)
Bangladesh	641	(630, 652)
India	531	(522, 541)
Maldives	395	(387, 404)
Nepal	606	(596, 617)
Pakistan	597	(586, 607)

**Table 3:** Adolescent pregnancy rate per 1000 ever-married women and 95% confidence interval (CI) in the six countries of South Asia by study variables.

	Afghanistan	Bangladesh	India	Maldives	Nepal	Pakistan
Study Variable	Rate (95% CI)	Rate (95% CI)	Rate (95% CI)	Rate (95% CI)	Rate (95% CI)	Rate (95% CI)
Community-level factor						
Type of residence						
Urban	667 (629, 704)	625 (591, 659)	517 (506, 529)	438 (240, 635)	588 (532, 644)	669 (604, 735)
Rural	723 (684, 762)	646 (611, 680)	534 (523, 546)	370 (188, 552)	625 (567, 683)	576 (515, 637)
Household-level factor						
Wealth index						
Richest	678 (640, 715)	548 (516, 580)	465 (454, 475)	1000 (701, 1299)	540 (486, 594)	627 (564, 690)
Richer	703 (664, 741)	582 (549, 615)	505 (494, 516)	308 (142, 473)	561 (506, 615)	588 (526, 649)
Middle	776 (735, 816)	659 (624, 694)	530 (518, 541)	400 (211, 589)	630 (571, 688)	599 (537, 660)
Poorer	673 (636, 711)	672 (637, 707)	520 (509, 531)	333 (161, 506)	642 (583, 700)	624 (561, 687)
Poorest	730 (691, 769)	722 (686, 759)	567 (556, 579)	385 (199, 570)	620 (562, 678)	564 (504, 624)
Sex of household head						
Female	345 (318, 372)	651 (617, 686)	503 (491, 514)	320 (151, 489)	603 (546, 660)	480 (425, 535)
Male	717 (678, 756)	640 (605, 674)	537 (525, 548)	500 (289, 711)	607 (550, 665)	607 (545, 670)
Individual-level factors						
Woman’s age in years						
15	237 (215, 260)	393 (366, 420)	186 (179, 192)	−	333 (291, 376)	286 (243, 328)
16	447 (416, 477)	496 (465, 526)	351 (342, 361)	−	321 (279, 362)	500 (443, 557)
17	636 (599, 673)	580 (548, 613)	440 (430, 451)	−	486 (435, 537)	506 (449, 563)
18	740 (700, 779)	641 (606, 675)	492 (481, 503)	−	670 (610, 730)	594 (533, 656)
19	811 (769, 852)	771 (733, 809)	614 (601, 626)	486 (277, 694)	730 (667, 793)	702 (635, 769)
Woman’s education						
Secondary or higher	704 (665, 742)	604 (570, 637)	528 (516, 539)	381 (196, 565)	604 (547, 661)	564 (504, 624)
Primary	586 (551, 621)	764 (726, 802)	559 (547, 570)	500 (289, 711)	632 (574, 690)	586 (524, 647)
No education	742 (703, 782)	689 (653, 725)	536 (525, 548)	−	591 (535, 647)	620 (557, 683)
Woman’s literacy level						
literate	669(631, 706)	631 (596, 665)	525(514, 537)	395 (207, 583)	608 (551, 665)	581 (520, 642)
Not literate	727(688, 766)	786 (747, 824)	553(541, 565)	−	600 (543, 657)	611 (548, 673)
Individual/social behavioral factors						
Access to radio						
Yes	707 (668, 745)	505 (474, 536)	499(488, 510)	429 (233, 624)	573 (517, 628)	481 (425, 536)
No	715 (676, 754)	656 (621, 691)	535(524, 547)	348 (172, 524)	654 (595, 714)	611 (548, 673)
Access to television						
Yes	706 (667, 744)	619 (585, 652)	527(516, 538)	390 (204, 577)	582 (526, 638)	578 (517, 639)
No	715 (676, 753)	681 (646, 717)	540(528, 551)	500 (289, 711)	650 (591, 709)	618 (555, 681)
Access to newspaper						
Yes	708 (669, 746)	464 (435, 493)	472(462, 483)	500 (289, 711)	590 (533, 646)	515 (458, 573)
No	712 (673, 750)	658 (623, 693)	550(538, 561)	250 (101, 399)	612 (555, 670)	608 (545, 670)
Heard of family planning on radio						
Yes	697 (659, 736)	438 (409, 466)	508(497, 519)	−	619 (561, 677)	556 (496, 615)
No	714 (675, 753)	642 (608, 677)	534(523, 546)	415 (222, 607)	601 (544, 658)	597 (535, 659)
Heard of family planning on television						
Yes	722 (683, 761)	597 (564, 630)	521(509, 532)	400 (211, 589)	591 (534, 647)	591 (529, 652)
No	708 (669, 747)	647 (613, 682)	543(531, 554)	382 (198, 567)	613 (556, 671)	597 (536, 659)
Heard of family planning in newspaper						
Yes	686 (648, 724)	688 (652, 723)	490(479, 501)	625 (389, 861)	393 (347, 439)	−
No	713 (674, 751)	640 (606, 675)	548(536, 560)	343 (168, 518)	625 (567, 683)	601 (539, 663)
Intention to use contraceptives						
Does not intend to use	640 (603, 676)	446 (417, 475)	633(621, 646)	406 (216, 597)	519 (466, 571)	564 (504, 624)
Currently using	950 (906, 995)	679 (643, 714)	533(521, 544)	200 (66, 334)	663 (603, 722)	1000 (920, 1080)
Intends to use later	850(808, 892)	620 (586, 654)	328(319, 337)	667 (423, 911)	595 (538, 651)	566 (506, 626)

Note: −, missing cell values.

**Table 4:** Unadjusted and adjusted odds ratios (OR) for factors associated with adolescent pregnancy in South Asia.

Study Variables	uOR (95% CI)	*p*-Value	aOR (95% CI)	*p*-Value
Country- and community-level factors				
Country of survey				
India	Reference		Reference	
Bangladesh	1.57 (1.41, 1.76)	<0.001	2.60 (2.26, 2.99)	<0.001
Afghanistan	2.17 (1.79, 2.63)		2.32 (1.91, 2.82)	
Maldives	0.57 (0.26, 1.26)		0.46 (0.19, 1.12)	
Nepal	1.36 (1.14, 1.63)		2.53 (2.06, 3.11)	
Pakistan	1.31 (1.08, 1.58)		1.43 (1.13, 1.80)	
Type of residence				
Urban	Reference			
Rural	1.03 (0.93, 1.14)	0.594		
Household-level factor				
Wealth index				
Richest	Reference		Reference	
Richer	1.04 (0.86, 1.25)	0.001	1.13 (0.92, 1.39)	<0.001
Middle	1.16 (0.97, 1.39)		1.34 (1.11, 1.62)	
Poorer	1.07 (0.89, 1.29)		1.27 (1.05, 1.54)	
Poorest	1.26 (1.06, 1.50)		1.50 (1.25, 1.81)	
Sex of household head				
Female	Reference		Reference	
Male	1.24 (1.11, 1.38)	<0.001	1.15 (1.03, 1.29)	0.015
Individual-level factors				
Woman’s age in years				
15	Reference		Reference	
16	1.92 (1.38, 2.66)	<0.001	2.15 (1.50, 3.08)	<0.001
17	2.75 (2.06, 3.67)		3.43 (2.52, 4.68)	
18	3.51 (2.65, 4.64)		4.60 (3.41, 6.19)	
19	5.35 (4.07, 7.03)		7.81 (5.82, 10.47)	
Woman’s education				
Secondary or higher	Reference			
Primary	1.30 (1.15, 1.48)	<0.001		
No education	1.39 (1.25, 1.54)			
Woman’s literacy level				
Literate	Reference			
Not literate	1.30 (1.18, 1.42)	<0.001		
Individual/social behavioral factors				
Access to radio				
Yes	Reference			
No	1.03 (0.91, 1.16)	0.66		
Access to television				
Yes	Reference			
No	1.14 (1.05, 1.23)	0.001		
Access to newspaper				
Yes	Reference		Reference	
No	1.46 (1.32, 1.61)	<0.001	1.35 (1.21, 1.50)	<0.001
Heard of family planning on radio				
Yes	Reference			
No	1.08 (0.96, 1.22)	0.177		
Heard of family planning on television				
Yes	Reference			
No	1.22 (1.12, 1.32)	<0.001		
Heard of family planning in newspaper				
Yes	Reference		Reference	
No	1.45 (1.32, 1.59)	<0.001	1.18 (1.06, 1.31)	0.003
Intention to use contraceptives				
Does not intend to use ever	Reference		Reference	
Currently using	0.80 (0.73, 0.89)	<0.001	0.75 (0.67, 0.83)	<0.001
Intends to use later	0.48 (0.43, 0.52)		0.40 (0.36, 0.44)	

CI: confidence interval; uOR = unadjusted odds ratio/univariate; aOR = adjusted odds ratio/multivariate.

## Data Availability

The DHS datasets can be found at https://dhsprogram.com/data/available-datasets.cfm (accessed on 10 June 2022).
